# The Role of Myokines and Adipokines in Hypertension and Hypertension-related Complications

**DOI:** 10.1038/s41440-019-0266-y

**Published:** 2019-05-27

**Authors:** Ken Chen, Mengdi Zhou, Xiaomei Wang, Shuang Li, Dachun Yang

**Affiliations:** 1 Department of Cardiology, The General Hospital of Western Theater Command, Chengdu, Sichuan 610083 China; 2 Department of Cardiology, Pidu District People’s Hospital, Chengdu, Sichuan 611730 China

**Keywords:** Adipokines, Myokines, Hypertension, Hypertension-related complications

## Abstract

The cross-talk between skeletal muscle and adipose tissue has been identified to play a key role in the regulation of blood pressure and the development of hypertension. The role of different adipokines and myokines in hypertension and hypertension-related complications remains unclear. In the present study, 98 hypertensive patients and 24 normotensive controls were recruited, and additional subgroup analyses of hypertension-related complications were also performed. The levels of the circulating bone-derived factors leptin, apelin, fractalkine, brain-derived neurotrophic factor (BDNF), leukemia inhibitory factor (LIF), myostatin, fatty-acid-binding protein 3 (FABP3), irisin, follistatin-related protein 1 (FSTL1), oncostatin M, fibroblast growth factor 21 (FGF21) and musclin were measured by a protein liquid chip assay. The circulating levels of BDNF and musclin were decreased, whereas the leptin and irisin levels were increased, in hypertensive patients compared with those in the control individuals. Further logistic analysis indicated that the irisin level was positively correlated with SBP and an independent predictor for hypertension after adjustment. In nonobese subjects, the concentrations of DKK1, BDNF and FSTL1 were decreased, whereas the concentrations of leptin and irisin were increased. Irisin and DKK1 might be associated with hypertension. Additional subgroup analyses showed that irisin is significantly associated with hypertension-related stroke. In conclusion, we found that increased irisin levels are associated with hypertension and hypertension-related stroke. These findings indicate that irisin may be involved in the pathophysiology of hypertension.

## Introduction

Hypertension causes a major share of the global disease burden [1]. Previous studies indicated that elevated blood pressure is responsible for approximately 60% of strokes and over 50% of ischemic heart disease [2]. As components of metabolic syndrome, obesity and hypertension are linked, and these two-coexisting cardiovascular disease risk factors increase cardiovascular disease morbidity and mortality [3]. Adipokines, which are various biologically active proteins produced by adipose tissue, are involved in obesity-related disorders including hypertension [4–6].

Moreover, increasing evidence has indicated that physical exercise is an effective nonpharmacological therapy for obesity and hypertension. The cross-talk among skeletal muscle, adipose tissue and the cardiovascular system caused by exercise plays a key role in protection against cardiovascular disease [7]. Previous studies have shown that skeletal muscle synthesizes and secretes multiple factors that exert beneficial effects on adipose tissue and the cardiovascular system that are called myokines [8, 9]. Myokines maintain fat stores, muscle mass and metabolic homeostasis [7]. However, little is known about the role of myokines in the regulation of blood pressure and the pathogenesis of hypertension. Thus, the present study was undertaken to examine the roles of adipokines and myokines in hypertension and hypertension-related complications by determining the circulating levels of adipokines and myokines.

## Methods

### Study design and setting

This study was an observational case-control study conducted in The General Hospital of Western Theater Command. The trial was registered in the Chinese Clinical Trial Registry (www.chictr.org.cn, identifier: ChiCTR1800016761) and received approval from the Ethics Committee of The General Hospital of Western Theater Command. Informed consent was received from all participants or the participants’ legal representatives.

### Subjects

For the present study, 122 hypertensive patients between 18 and 70 years of age were recruited retrospectively from the Department of Cardiology in The General Hospital of Western Theater Command between Oct. 20, 2017, and Apr. 11, 2018. The patients had a mean blood pressure (BP) on the reference arm with a systolic BP ≥140 and/or a diastolic BP ≥90 and were diagnosed with hypertension. The exclusion criteria were as follows: (1) secondary hypertension (2), acute myocardial infarction and acute stroke (less than 3 months) (3), mental and physical disability (4), combined severe disease with a life expectancy of less than 1 year, and (5) refusal to join the project. In addition, 30 subjects without hypertension were collected from the same department in The General Hospital of Western Theater Command during the same period to serve as controls. The exclusion criteria for the control group were the same as those for the hypertension group.

Each subject’s history of hypertension-related complications (including coronary artery disease, arrhythmia, stroke, peripheral vascular disease, chronic kidney disease, diabetes and hyperlipidemia) was investigated retrospectively via medical records. Coronary artery diseases in this study included acute coronary syndrome and chronic ischemic syndrome diagnosed by previous clinical manifestations, electrocardiogram evaluation and/or coronary angiography [10–12]. A history of arrhythmia was identified by previous electrocardiogram evaluation and a diagnosis of tachycardia, sinus bradycardia, sick sinus syndrome, extrasystole, supraventricular tachycardia, ventricular tachycardia, atrial flutter, atrial fibrillation or heart block. Previous instances of stroke were diagnosed by brain imaging with computed tomography (CT) or magnetic resonance imaging (MRI) and included ischemic or hemorrhagic events and transient ischemic attack (TIA) [13, 14]. Peripheral vascular diseases were diagnosed by carotid ultrasound or Doppler examination of the arterial limb. Chronic kidney disease was defined as either microalbuminuria or an estimated glomerular filtration rate (eGFR) below 60 ml/min/1.73 m^2^ [15]. Diabetes was defined as having a fasting plasma glucose level above 7.1 mmol/l [16]. Hyperlipidemia in the Chinese population was defined as total cholesterol (TC) >5.17 mmol/l or/and plasma triglyceride (TG) >2.3 mmol/l. Moreover, obesity for the Chinese population was defined as having a BMI (body mass index) greater than 28 kg/m^2^.

### Biochemical assays and adipokine/myokine measurements

Peripheral venous blood samples were collected between 6 and 7 a.m. after overnight fasting, kept at room temperature for clotting and centrifuged at 3,000 × g for 15 min to obtain serum. Serum fasting blood glucose (FBG), triglyceride (TG), total cholesterol (TC), high-density lipoprotein cholesterol (HDL-C), low-density lipoprotein cholesterol (LDL-C), creatinine (Cre) and blood urea nitrogen (BUN) levels were checked by an automatic chemistry analyzer (Beckman Coulter, Inc., Brea, CA, USA). The levels of the adipokines and myokines leptin, apelin, fractalkine, BDNF, LIF, myostatin, FABP3, irisin, FSTL1, oncostatin M, FGF21 and musclin in the serum were measured by an adipokine- and myokine-specific Luminex bead-based multiplex detection system (Merck Millipore, Darmstadt, Germany).

### Statistical analyses

Statistical analyses were conducted with SPSS 22.0 statistics software (IBM SPSS Inc., Chicago, IL, USA). The data are expressed as the mean ± SD, the median (IQR 25–75) or percentages as appropriate. Comparisons within two groups were made by independent t-test, Mann–Whitney U test or *Χ*^2^ test, for continuous variables, nonparametric variables or proportions, respectively. Univariate logistic analysis was used to select the covariates in the multivariate model, while multivariable logistic regression analysis was performed to evaluate the association between the serum bone-derived factors and hypertension after adjusting for other potential confounders. The results are shown here with odds ratios and 95% confidence intervals (CIs). Correlation analysis was performed by Pearson analysis for parametric variables and Spearman analysis for nonparametric variables. A *P* value <0.05 indicated statistical significance.

## Results

### Characteristics of the participants included in the study

The baseline characteristics of all participants are summarized in Table 1. The hypertensive patients were significantly older than the control participants. The levels of BMI, SBP, DBP, FBG and HbA1c were higher in the hypertensive group than in the participants without hypertension, whereas the HDL-C and eGFR levels, exercise frequency and exercise duration per week of the hypertensive patients were lower (*P* < 0.05) than those in the controls.

**Table 1:** Baseline Characteristics of the participants in normotensive and hypertensive group

Parameters	NT (*n* = 24)	HT (*n* = 98)	*P* Value
Gender (M/F)	12/12	49/49	1.0
Age (years)	53.5 (44.5–61.25)	63 (55.25–68)*	<0.0001
Height (cm)	162.21 ± 9.39	158.95 ± 8.04	0.088
Body weight (kg)	60.94 ± 10.48	64.69 ± 11.11	0.137
BMI (kg/m^2^)	23.28 ± 3.61	25.53 ± 3.44*	0.005
SBP (mmHg)	112.88 ± 10.39	177.44 ± 21.29*	<0.0001
DBP (mmHg)	79.71 ± 7.68	97.77 ± 12.69*	<0.0001
FBG (mmol/l)	4.73 ± 0.78	6.22 ± 2.96*	0.016
HbA1c (mg/dl)	5.45 ± 0.47	6.24 ± 1.76*	0.033
TG (mmol/l)	1.52 ± 1.04	2.04 ± 1.57	0.22
TC (mmol/l)	4.52 ± 1.08	4.23 ± 1.05	0.126
HDL-C (mmol/l)	1.41 ± 0.41	1.26 ± 0.30*	0.04
LDL-C (mmol/l)	2.53 ± 0.83	2.41 ± 0.87	0.557
Cre (µmol/l)	68.0 ± 13.98	84.94 ± 56.01	0.145
BUN (mmol/l)	5.72 ± 1.87	6.12 ± 3.56	0.589
eGFR (ml/min/1.73 m^2^)	108.22 ± 20.66	91.74 ± 25.64*	0.004
Exercise frequency (per week)	5.5 (5–7)	3 (0–6)*	0.005
Exercise duration (min/week)	43.33 ± 33.61	23.98 ± 23.16*	0.001
Alcohol consumption (g/day)	0 (0–0)	0 (0–0)	0.279
Smoking (cigarettes per day)	6.42 ± 10.89	5.72 ± 13.25	0.811

Values are provided as mean ± SD, median (IQR 25–75) or percentages, as appropriate

*NT* normotensive subjects, *HT* hypertensive subjects, *BMI* body mass index, *SBP* systolic blood pressure, *DBP* diastolic blood pressure, *HDL-C* high-density lipoprotein cholesterol, *LDL-C* low-density lipoprotein cholesterol, *TC* total cholesterol, *TG* triglyceride, *FBG* fasting blood glucose, *Cre* creatinine, *BUN* blood urea nitrogen, *eGFR* glomerular filtration rate

*P < 0.05, vs. NT group. *P* values are from two-tailed tests

### Circulating levels of adipokines and myokines in hypertensive patients and controls

**Fig. 1:**
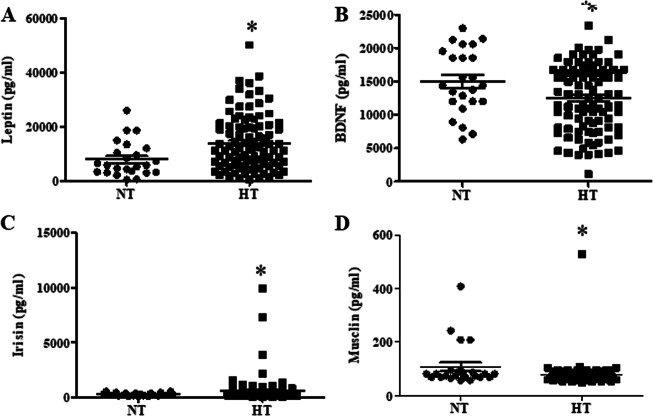
Serum concentrations of leptin (**a**), BDNF (**b**), irisin (**c**) and musclin (**d**) in the hypertensive group and controls. The adipokine and myokine levels were measured by a protein liquid chip assay (*P < 0.05 vs. control; *n* = 24 in the control and *n* = 98 in the hypertensive group)

Further studies determined the levels of circulating adipokines and myokines by a protein liquid chip assay. The levels of circulating BDNF and musclin derived from hypertensive participants were considerably lower than those derived from the controls, whereas the leptin and irisin concentrations were higher in hypertensive participants than in controls (Fig. 1).

### Association of serum adipokines and myokines with hypertension

Logistic regression analysis was performed to test the associations of BDNF, musclin, leptin and irisin with hypertension. The significant covariates for hypertension determined by univariate analysis were included in the multivariate model; these were age, BMI, FBG, HbA1c, HDL-C level, eGFR, exercise frequency and exercise duration (Supplemental Table 1). After adjustment for BMI, FBG, HbA1c, HDL-C level, eGFR, exercise frequency and exercise duration, the first model showed that irisin was associated with hypertension (OR = 1.004, 95% CI: 1.00–1.008; *P* < 0.05). Additionally, a complete model including all the covariates was determined; this model also showed that a high irisin level was an independent predictor for hypertension (irisin: OR = 1.004, 95% CI: 1.000–1.009; *P* < 0.05), as shown in Table 2.

**Table 2:** Association of the circulating myokines and adiokines levels with hypertension risks based on the multiple logistic regression analysis

Model 1		
Leptin (pg/ml)	1.00 (1.00–1.00)	0.012
BDNF (pg/ml)	1.00 (1.00–1.00)	0.332
Irisin (pg/ml)	1.004 (1.00–1.008)*	0.049
Musclin (pg/ml)	0.993 (0.986–1.001)	0.068
*Model 2*		
Leptin (pg/ml)	1.00 (1.000–1.001)*	0.018
BDNF (pg/ml)	1.00 (1.00–1.00)	0.173
Irisin (pg/ml)	3.045 (0.000–23420754.549)	0.891
Musclin (pg/ml)	0.987 (0.974–1.001)	0.063

Adjusted odds ratio (OR) and 95% confident intervals (CI) were performed by the multiple logistic regression analysis

Model 1: adjusted for age, gender, BMI, FBG, HbA1c, HDL-c, eGFR, exercise frequency and exercise duration

Model 2: full model, adjusted for TC, TG, LDL-C, Cre, BUN, alcohol and tobacco consumption based on model 2 *P < 0.05

We then analyzed the relationships between the serum levels of leptin, BDNF irisin and musclin with blood pressure in the hypertensive group and controls and irisin was positively correlated with SBP in all subjects (*r* = 0.18, *P* < 0.05, Table 3).

**Table 3:** The correlation between the serum myokines and adipokines and blood pressure in normotensive and hypertensive groups

Parameters	SBP (mmHg)			DBP (mmHg)		
	Total	NT	HT	Total	NT	HT
Leptin (pg/ml)	0.278^a^	−0.064	0.162	0.121	−0.142	−0.001
BDNF (pg/ml)	−0.113	−0.075	0.028	−0.021	0.192	0.081
Irisin (pg/ml)	0.180^a^	0.162	−0.131	0.061	−0.328	0.039
Musclin (pg/ml)	−0.159	0.323	−0.040	−0.015	0.296	0.049

^a^*P* < 0.05 shows significant correlation

### Association of serum adipokines and myokines with hypertension in nonobese subjects

**Fig. 2:**
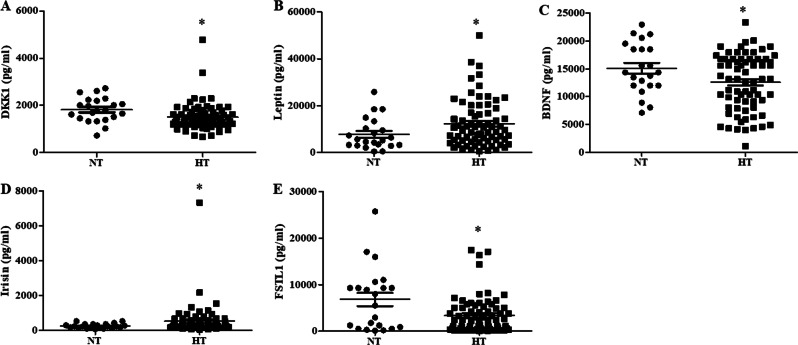
The serum concentrations of DKK1 (**a**), leptin (**b**), BDNF (**c**), irisin (**d**) and FSTL1 (**e**) in nonobese subjects with or without hypertension. The adipokine and myokine levels were measured by a protein liquid chip assay (*P < 0.05 vs. control; *n* = 22 normotensive subjects and *n* = 73 hypertensive subjects)

Since circulating adipokine and myokine levels are associated with adipogenesis and obesity [17, 18], we then analyzed the levels of adipokines and myokines in nonobese subjects with or without hypertension and the association of adipokines and myokines with hypertension. The baseline characteristics of the nonobese subjects are summarized in Supplemental Table 2. The concentrations of DKK1, BDNF and FSTL1 were lower, whereas the concentrations of leptin and irisin were higher, in hypertensive patients than in normotensive subjects (Fig. 2). In addition, logistic regression analysis was also performed to test the associations of DKK1, BDNF, FSTL1, leptin and irisin with hypertension, and irisin was significantly associated with hypertension (OR = 1.013, 95% CI: 1.002–1.025; *P* < 0.05) after adjusting for age, gender, height, BMI, FBG, HbA1c, TC level, Cre level, eGFR, exercise frequency and exercise duration, while DKK1 was associated with hypertension (OR = 0.995, 95% CI: 0.99–0.999; *P* < 0.05) in a complete model including all the covariates (Table 4).

**Table 4:** Association of the circulating myokines and adiokines levels with hypertension risks based on the multiple logistic regression analysis in nonobese subjects

Model 1		
DKK1 (pg/ml)	0.998 (0.995–1.000)	0.077
Leptin (pg/ml)	1.000 (1.000–1.000)	0.049
BDNF (pg/ml)	1.000 (1.000–1.000)	0.631
Irisin (pg/ml)	1.013 (1.002–1.025)*	0.018
FSTL1 (pg/ml)	1.000 (1.000–1.000)	0.258
Model 2		
DKK1 (pg/ml)	0.995 (0.990–0.999)*	0.03
Leptin (pg/ml)	1.000 (1.000–1.001)	0.159
BDNF (pg/ml)	1.000 (1.000–1.001)	0.862
Irisin (pg/ml)	1.241 (0.000–3.554 × 10^12^)	0.988
FSTL1 (pg/ml)	1.000 (0.999–1.000)	0.345

Adjusted odds ratio (OR) and 95% confident intervals (CI) were performed by the multiple logistic regression analysis

Model 1: adjusted for age, gender, height, BMI, FBG, HbA1c, TC, Cre, eGFR, exercise frequency and exercise duration

Model 2: full model, adjusted for body weight, TG, HDL-C, LDL-C, BUN, alcohol and tobacco consumption based on model 2 *P < 0.05

The relationships between the serum levels of DKK1, BDNF, FSTL1, leptin and irisin with blood pressure in the hypertensive group and controls were analyzed. As listed in Table 5, the serum leptin and irisin concentrations were positively associated with SBP in nonobese subjects (*r* = 0.251 and 0.261, *P* < 0.05).

**Table 5:** The correlation between the serum myokines and adipokines and blood pressure in nonobese subjects with or without hypertension

Parameters	SBP (mmHg)			DBP (mmHg)		
	Total	NT	HT	Total	NT	HT
DKK1 (pg/ml)	−0.134	0.007	0.117	−0.123	−0.122	0.011
BDNF (pg/ml)	−0.152	−0.124	0.065	0.006	0.121	0.140
FSTL1 (pg/ml)	−0.198	−0.298	0.200	−0.142	−0.169	0.066
Leptin (pg/ml)	0.251^a^	−0.053	0.184	0.084	−0.12	−0.007
Irisin (pg/ml)	0.261^a^	0.193	−0.01	0.103	−0.333	−0.002

^a^*P* < 0.05 shows significant correlation

### Association of serum adipokines and myokines with hypertension-related complications in hypertensive subjects

**Fig. 3:**
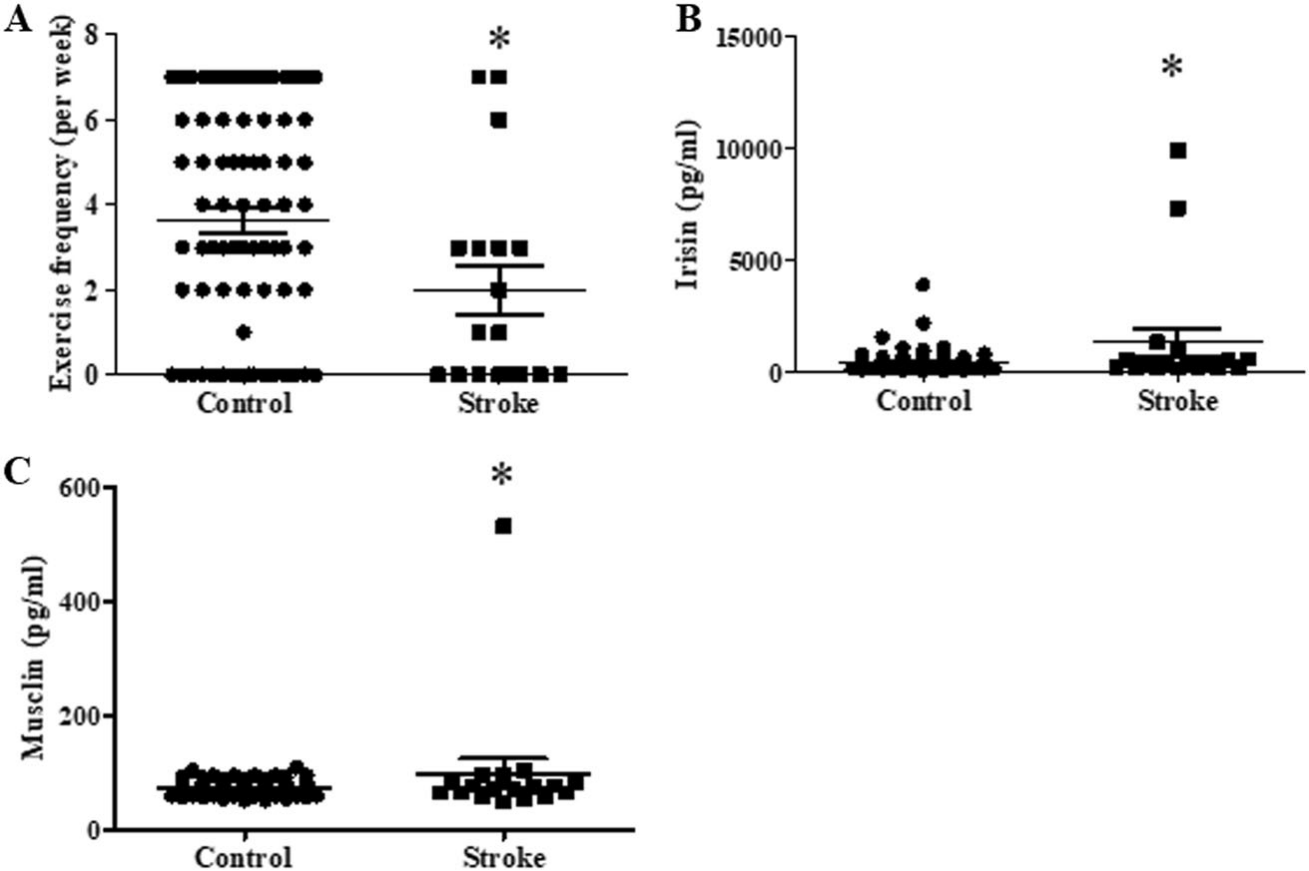
The exercise frequency (**a**) and serum concentrations of irisin (**b**) and musclin (c) in hypertension-related stroke patients. (*P < 0.05 vs. control; *n* = 80 without stroke and *n* = 18 with stroke)

Further subgroup analysis was conducted to determine the role of several adipokines and myokines in hypertension-related complications in hypertensive subjects. The percentages of hypertensive patients with different hypertension-related complications are listed in Supplemental Table 3. The three leading complications were coronary artery disease, arrhythmia and diabetes. We then analyzed the differences in adipokine and myokine levels in patients with different hypertension-related complications. No significant differences in the adipokine and myokine levels and clinical parameters were found between hypertensive patients and hypertensive patients with complications such as coronary artery disease, arrhythmia, peripheral vascular disease, diabetes and hyperlipidemia (data not shown). In contrast, exercise frequency was significantly decreased in the hypertensive patients with stroke, and the irisin and musclin concentrations were higher in the hypertension-related stroke patients than those in the hypertensive participants (Fig. 3). On average, the serum of patients with hypertension contained 456.9 ± 57.92 pg/ml irisin and 72.05 ± 1.41 pg/ml musclin, while the serum of patients with hypertension-related stroke contained 1333 ± 637.8 pg/ml irisin and 97.45 ± 25.62 pg/ml musclin.

Moreover, logistic regression analysis was performed to test the association of irisin and musclin with stroke in hypertensive subjects. Only irisin was significantly associated with hypertension-related stroke (OR = 1.001, 95% CI: 1.00–1.001; *P* < 0.05) after adjusting for age, gender and exercise frequency (Table 6), and irisin and musclin were not correlated with SBP or DBP in the subgroup with stroke (Supplemental Table 4).

**Table 6:** Association of the circulating myokines and adipokines levels with stroke in the hypertensive subjects based on the multiple logistic regression analysis

Parameters	Adjusted OR (95% CI)	*P* Value
Irisin (pg/ml)	1.001 (1.00–1.001)*	0.04
Musclin (pg/ml)	1.016 (0.991–1.041)	0.215

Adjusted odds ratio (OR) and 95% confident intervals (CI) were performed by the multiple logistic regression analysis

Adjusted for age, gender and exercise frequency *P < 0.05

## Discussion

Obesity has been recognized as one of the major risk factors for the development of hypertension [5, 19], and physical exercise to reduce blood pressure in individuals with hypertension is widely recommended by international guidelines [20–22]. Adipose tissue accumulation and skeletal muscle loss are both independent risk factors for the development of hypertension [19, 23, 24]. Therefore, the cross-talk between skeletal muscle and adipose tissue should be involved in the regulation of blood pressure and the development of hypertension. Adipokines released from adipose tissue and myokines released from skeletal muscle may participate in the reciprocal regulation of the adipose-muscular axis and exert different effects in hypertension.

Our present study revealed that adipokines and myokines might be associated with hypertension. In this study, we found that the circulating levels of BDNF and musclin were decreased, whereas leptin and irisin levels were increased, in hypertensive patients compared with those in the control individuals. Further logistic analysis indicated that the irisin level was an independent predictor for hypertension after adjusting for other factors. Moreover, we found that the DKK1, BDNF and FSTL1 levels were lower, whereas the concentrations of leptin and irisin were higher, in nonobese hypertensive patients than in normotensive subjects. Irisin was positively correlated with SBP and an independent predictor for hypertension in nonobese subjects as well.

Irisin, a type I membrane protein encoded by the Fndc5 gene and secreted by skeletal muscle after exercise, participates in mitochondrial biogenesis and adipose tissue browning and improves obesity and glucose homeostasis [25, 26]. Some studies have provided evidence that irisin is associated with the regulation of blood pressure and hypertension. Data from an animal model of hypertension showed that irisin lowers blood pressure, which was ascribed to AMPK-induced eNOS phosphorylation and increased NO release in endothelial cells [27], indicating that irisin contributes to antihypertension. However, controversy has emerged regarding the association between irisin and blood pressure in various clinical studies. A study carried out by Celik et al. did not discover a significant difference in irisin levels between untreated hypertensive patients and controls [28], while no significant difference in serum irisin levels was found among severely preeclamptic patients, mildly preeclamptic patients and normal controls [29]. Despite a report revealing a negative association between serum irisin and blood pressure [29], a cross-sectional study including 532 patients with chronic kidney disease provided evidence in favor of the opposite effect of irisin on diastolic blood pressure [30]. Our results showed that the levels of irisin in hypertensive individuals were higher than those in normotensive controls, which was independently associated with hypertension after adjustment.

However, it is not known whether the elevation in irisin levels was the cause or consequence of hypertension in our study. Since hypertension is a well-known systemic inflammation- and oxidative stress-related disease, previous studies have shown that decreasing inflammation and reactive oxygen species levels might, in part, have a beneficial effect in controlling hypertension [31–33]. Previous studies showed a correlation between irisin levels and the levels of inflammatory factors [34, 35] and that oxidative stress might elevate the circulating irisin level [36]. Moreover, we have also presented data showing that irisin is an endogenous anti-inflammatory and antioxidative hormone that prevents pulmonary and cardiac injury [37, 38]. Therefore, we inferred that irisin elevation in hypertensive patients might be a response to hypertension-associated inflammation and oxidative stress that provides feedback to maintain homeostasis. The underlying mechanisms of this function remain largely unknown, and further research is needed to expand knowledge of these mechanisms.

Moreover, hypertension-related complications, including coronary heart disease, stroke, peripheral arterial disease and chronic kidney disease, are clinical outcomes resulting from elevated blood pressure [39, 40]. Further analysis revealed that only irisin, but not other adipokines and myokines, was significantly associated with hypertension-related stroke and not associated with other complications. Although an animal study from Li et al. noted decreased plasma irisin concentrations in cerebral ischemia-injured mice [41], our data showed that the level of circulating irisin was increased in hypertensive subjects with stroke compared to that in control subjects. Several studies have found that irisin might protect against stroke in rodent models or patients [41–43]. Therefore, we inferred that an elevation in the irisin concentration in patients with hypertension-related stroke might be a protective response to hypertensive target organ damage.

Our study has several limitations. First, because it is a cross-sectional study, the cause-effect relationship between adipokines or myokines and the diseases could not be determined. Second, the analysis included only a limited number of serum samples. We should be able to expand the sample size to establish a causative relationship between changes in the levels of adipokines or myokines and hypertension and its related complications. Third, the roles and the mechanisms of irisin elevation in patients with hypertension and hypertension-related stroke remain unclear and need to be determined in the future.

In conclusion, our present study found that increased circulating irisin was associated with hypertension and hypertension-related stroke. These findings indicate that adipokines or myokines may be involved in the pathophysiology of hypertension and call for further basic and clinical investigation.

## Supplementary information

Supplemental Table 1

Supplemental Table 2

Supplemental Table 3

Supplemental Table 4
