# Supplementary material for: The Role of Myokines and Adipokines in Hypertension and Hypertension-related Complications
Source: Hypertens Res. 2019 May 27;42(10):1544–51. doi: 10.1038/s41440-019-0266-y (PMC8076012; doi:10.1038/s41440-019-0266-y)
Supplement: Supplementary file 1 — Supplemental Table 1 [file 41440_2019_266_MOESM1_ESM.docx]

**Supplemental table 1.** Univariate analysis of significant covariates for hypertension

| Parameter | Adjusted OR (95%CI) | P Value |
| --- | --- | --- |
| Age (years) | 1.084 (1.037-1.132) * | <0.0001 |
| BMI (kg/m^2^) | 1.213 (1.054-1.391) * | 0.007 |
| FBG (mmol/l) | 4.278 (1.949-9.39) * | 0.0003 |
| HbA1c (mg/dl) | 3.729 (1.578-8.813) * | 0.003 |
| HDL-C (mmol/l) | 0.259 (0.068-0.979) * | 0.046 |
| eGFR (ml/min/1.73m^2^) | 0.972 (0.953-0.992) * | 0.006 |
| Exercise frequency (per week) | 0.744 (0.608-0.912) * | 0.004 |
| Exercise duration (min/week) | 0.974 (0.956-0.993) * | 0.006 |
| Exercise duration (min/week) | 0.974 (0.956-0.993) * | 0.006 |
