# Supplementary material for: The Role of Myokines and Adipokines in Hypertension and Hypertension-related Complications
Source: Hypertens Res. 2019 May 27;42(10):1544–51. doi: 10.1038/s41440-019-0266-y (PMC8076012; doi:10.1038/s41440-019-0266-y)
Supplement: Supplementary file 2 — Supplemental Table 2 [file 41440_2019_266_MOESM2_ESM.docx]

**Supplemental table 2** Baseline Characteristics of the participants in non-obese subjects with or without hypertension

| Parameters | NT (n=22) | HT (n=73) | P Value |
| --- | --- | --- | --- |
| Gender (M/F) | 11/11 | 36/37 | 1.0 |
| Age (years) | 52 (44.5-59.25) | 63 (58.5-68) * | <0.0001 |
| Height (cm) | 163.00±9.26 | 159.03±7.02 * | 0.034 |
| Body weight (kg) | 60.11±10.56 | 60.96±8.82 | 0.708 |
| BMI (kg/m^2^) | 22.69±3.10 | 24.02±2.43* | 0.038 |
| SBP (mmHg) | 113.00±10.83 | 176.53±20.52* | <0.0001 |
| DBP (mmHg) | 79.41±7.74 | 96.71±13.50* | <0.0001 |
| FBG (mmol/l) | 4.59±0.57 | 6.18±3.22* | 0.024 |
| HbA1c (mg/dl) | 5.40±0.43 | 6.27±1.96* | 0.042 |
| TG (mmol/l) | 1.57±1.07 | 1.78±1.27 | 0.467 |
| TC (mmol/l) | 4.70±0.93 | 4.18±1.00* | 0.031 |
| HDL-C (mmol/l) | 1.44±0.42 | 1.30±0.30 | 0.074 |
| LDL-C (mmol/l) | 2.65±0.75 | 2.39±0.87 | 0.202 |
| Cre (µmol/l) | 68.83±14.29 | 84.94±56.01* | 0.035 |
| BUN (mmol/l) | 5.63±1.83 | 6.42±3.91 | 0.363 |
| eGFR (ml/min/1.73m^2^) | 107.6±21.08 | 93.93±26.13* | 0.027 |
| Exercise frequency (per week) | 6 (4.75-7) | 3 (0-6) * | 0.008 |
| Exercise duration (min/week) | 44.55±34.91 | 24.18±21.76* | 0.001 |
| Alcohol consumption (g/day) | 3.18±10.41 | 6.85±17.86 | 0.36 |
| Smoking (cigarettes per day) | 0 (0-20) | 0 (0-2.5) | 0.216 |

Values are provided as mean ± SD, median (IQR 25-75) or percentages, as appropriate.

NT, normotensive subjects; HT, hypertensive subjects; BMI, body mass index; SBP or DBP, systolic and diastolic blood pressure; HDL-C, high-density lipoprotein cholesterol; LDL-C, low-density lipoprotein cholesterol; TC, total cholesterol; TG, triglyceride; FBG, fasting blood glucose; Cre, creatinine; BUN, blood urea nitrogen; eGFR, glomerular filtration rate.

*P<0.05, vs. NT group. P values are from two-tailed tests.
