# Supplementary material for: The Role of Myokines and Adipokines in Hypertension and Hypertension-related Complications
Source: Hypertens Res. 2019 May 27;42(10):1544–51. doi: 10.1038/s41440-019-0266-y (PMC8076012; doi:10.1038/s41440-019-0266-y)
Supplement: Supplementary file 3 — Supplemental Table 3 [file 41440_2019_266_MOESM3_ESM.docx]

**Supplemental table 3.** The prevalence of hypertension-related complications in the hypertensive subjects

| Target Organ Damages | N (%) |
| --- | --- |
| Coronary artery disease | 51 (52.04%) |
| Arrhythmia | 30 (30.61%) |
| Stroke | 18 (18.37%) |
| Peripheral vascular disease | 9 (9.18%) |
| Chronic kidney disease | 4 (4.08%) |
| Diabetes | 22 (22.45%) |
| Hyperlipidemia | 18 (18.37%) |
