# Supplementary material for: The Role of Myokines and Adipokines in Hypertension and Hypertension-related Complications
Source: Hypertens Res. 2019 May 27;42(10):1544–51. doi: 10.1038/s41440-019-0266-y (PMC8076012; doi:10.1038/s41440-019-0266-y)
Supplement: Supplementary file 4 — Supplemental Table 4 [file 41440_2019_266_MOESM4_ESM.docx]

**Supplemental table 4** The correlation between the serum irisin and blood pressure in stroke subjects

| Parameters | SBP (mmHg) | DBP (mmHg) |
| --- | --- | --- |
| Irisin (pg/ml) | -0.100 | 0.240 |
| Musclin | -0.002 | 0.103 |

*P<0.05 shows significant correlation.
